# Broad and narrow personality traits as markers of one-time and repeated suicide attempts: A population-based study

**DOI:** 10.1186/1471-244X-8-15

**Published:** 2008-03-06

**Authors:** Jelena Brezo, Joel Paris, Martine Hébert, Frank Vitaro, Richard Tremblay, Gustavo Turecki

**Affiliations:** 1 McGill Group for Suicide Studies, Douglas Hospital Research Center, Montreal, Canada; 2 Department of Psychiatry, McGill University, Montreal, Canada; 3 GRIP, University of Montreal, Montreal, Canada; 4 Department of Sexology, University of Quebec, Montreal, Canada

## Abstract

**Background:**

Studying personality traits with the potential to differentiate between individuals engaging in suicide attempts of different degrees of severity could help us to understand the processes underlying the link of personality and nonfatal suicidal behaviours and to identify at-risk groups. One approach may be to examine whether narrow, i.e., lower-order personality traits may be more useful than their underlying, broad personality trait dimensions.

**Methods:**

We investigated qualitative and quantitative differences in broad and narrow personality traits between one-time and repeated suicide attempters in a longitudinal, population-based sample of young French Canadian adults using two multivariate regression models.

**Results:**

One broad (Compulsivity: OR = 2.0; 95% CI 1.2–3.5) and one narrow personality trait (anxiousness: OR = 1.1; 95% CI 1.01–1.1) differentiated between individuals with histories of repeated and one-time suicide attempts. Affective instability [(OR = 1.1; 95% CI 1.04–1.1)] and anxiousness [(OR = .92; 95% CI .88–.95)], on the other hand, differentiated between nonattempters and one-time suicide attempters.

**Conclusion:**

Emotional and cognitive dysregulation and associated behavioural manifestations may be associated with suicide attempts of different severity. While findings associated with narrow traits may be easier to interpret and link to existing sociobiological theories, larger effect sizes associated with broad traits such as Compulsivity may be better suited to objectives with a more clinical focus.

## Background

Personality traits may be of value as correlates [1,2], predictors [3], endophenotypes [4,5], and targets of health interventions in the context of a number of psychiatric phenotypes [6,7], including suicidality. There is a growing recognition that the extent of involvement of personality traits with such phenotypes may depend on the position that these traits occupy in personality hierarchy. A distinction has been made between the more specific levels of personality structure, consisting of narrow or lower-order personality traits, and the higher-order, broader dimensions to which they contribute [8,9].

In an ongoing debate on the merits of these two personality levels, it has been recognized that narrow personality traits [10,11] allow higher predictive accuracy and a better understanding of the relevant etiological mechanisms [12]. This is, in part, because broad personality traits consist only of shared variance of contributing narrow personality traits, while the latter also have unique variance that may be of relevance to some risk behaviours [10,13]. Consequently, an association between an outcome and a narrow personality trait may occur even in the absence of a similar association with a related, broad personality trait [12]. Nevertheless, broad personality factors may have greater "bandwidth" and be easier to measure in some cases [13].

When it comes to suicide attempts, an important antecedent of suicide completions [14,15], personality traits may be involved in a dosage-dependent fashion, correlating positively with phenotypic severity [16]. Namely, individuals with histories of repeated suicide attempts may also have more extreme personality profiles than individuals who have attempted only once. Nevertheless, despite the clinical importance of understanding repeated suicide attempts, research on the associated risk factors is still scarce [16,17], especially when it comes to the evidence concerning personality traits. In general, suicide attempters have been observed to have higher levels of aggression, anxiety, neuroticism, extroversion, impulsivity, and psychoticism [1]. In an earlier study in the same sample [18], focusing on suicide attempters as a group, we examined narrow personality trait correlates of previous suicide attempts and of current, serious suicidal ideation. We found small, although significant, contribution of an externalizing personality trait of conduct problems to both of these suicidal phenotypes.

While studies focusing on suicide attempters are useful, they cannot indicate whether there are specific personality traits that may serve as markers of possibly distinct subpopulations of suicide attempters [16], such as one-time and repeated suicide attempters, for example. Using a comparative approach, we investigated qualitative and quantitative differences between broad and narrow personality trait correlates of repeated and one-time suicide attempt histories. We operationalized qualitative differences as those encompassing traits that are dysregulated in selected subgroups of suicide attempters; Quantitative differences, on the other hand, would pertain to traits that are dysregulated across suicide attempter subgroups although, possibly, to a somewhat different extent. We assessed two levels of personality traits (17 narrow and 4 broad personality dimensions) using the Diagnostic Assessment of Personality Pathology-Basic Questionnaire (DAPP-BQ), an instrument with satisfactory psychometric properties [19,20]. Analyses were conducted in a sizable, longitudinal, population-based sample of young French Canadian adults. On the basis of previous evidence, we hypothesized that three broad DAPP-BQ dimensions-Emotional Dysregulation [16,21,22], Dissocial Behavior [23]; and Inhibition [24,25], and associated narrow traits, will be highest in our multiple suicide attempters relative to one-time attempters and nonattempters.

## Methods

### Study participants and data collection

Our sample consisted of 21–24 year-old adults. These individuals have been followed since their last kindergarten year in one of the French-speaking public schools in Quebec [26,27]. The total initial sample comprised 957 boys and 946 girls randomly selected to represent urban and rural Quebec. To reduce cultural heterogeneity, only children whose parents were born in Canada and whose mother tongue was French were included in this cohort. The majority of children (89%) were non-Hispanic Whites. The remainder reported being of Native Indian, Asian, Black, and White Hispanic origin. Only participants who had provided complete data on our personality measures in adulthood (n = 1094) were included. The remainder provided incomplete questionnaire data (n = 59); were disabled, or died by suicide or from other causes (n = 13); could not be (n = 12) or were never contacted (n = 11); could not be traced (n = 99); refused to participate (n = 196); lived in a remote area (18); or were missing for unknown reasons (n = 498).

The data were obtained using a combination of interviews and self-reports. The study was approved by the research ethics boards of the University of Montreal and McGill University. Written informed consent was obtained from all subjects.

### Instruments

#### 1. Sociodemographic factors in childhood and adulthood

Family circumstances surrounding participants as children, consisting of various indices of parental sociodemographic status and living arrangements, were obtained yearly from 6 to 12 years of age.

#### 2. Traumatic events

##### a. Childhood physical violence (Revised Conflict Tactics Scales) [28,29]

Using self-reports on 32 questions, this scale assesses childhood incidences of nonviolent discipline, psychological aggression, and physical assault in parent-child and other family relationships. In this study, we selected 14 questions dealing with severe and very severe physical aggression, abuse, and injuries perpetrated by each parent (biological, adaptive, or any person in a parental role) against the respondent as a child.

##### b. Childhood sexual violence [30,31]

This instrument measures incidences of sexual violence since birth up to the age of 18. Questions ask about abusive sexual acts involving some degree of physical contact (fondling or sexual – vaginal, anal, or oral – relationship imposed by threats or use of force) and perpetrated by immediate family members, school peers/personnel, short/long-term romantic acquaintances, or strangers.

##### c. Physical partner violence (Revised Conflict Tactics Scales) [29]

Using self-reports, this scale assesses incidences of psychological and physical aggression, sexual coercion, and physical injuries. In order to assess violence perpetrated by the current or partners of the previous five years against the respondent, we selected 11 questions inquiring about acts of severe and very severe aggression and injuries.

##### d. Yearly calendars – stressful life events

Using yearly calendars, participants were also asked about atypical/severe stressful events related to finances, school or work, death of a close person, illness or serious injury to self or others, parental separation/divorce, partner/children difficulties, and "other" sources of stress. Most stress items were adapted from an existing instrument [32].

#### 3. Psychiatric diagnoses

##### a. Diagnostic Interview Schedule for adults and children [33]

Psychiatric diagnoses in this study were based on DIS interviews using DSM-III-R criteria. In terms of adult diagnoses, in both mothers and participants, we focused on substance abuse/dependence involving drugs, alcohol, and nicotine; major depressive episodes, mania, and dysthymia; generalized anxiety, panic and phobias. Substance misuse phenotypes were considered individually and mood and anxiety disorders as groups in both adolescence and adulthood. Adolescent diagnoses were assessed using parental and self-reports and similarly grouped into disruptive (attention-deficit-hyperactivity, oppositional-defiant, and conduct disorders), anxiety (simple and social phobias, separation anxiety, panic, avoidant, overanxious, and generalized anxiety disorder), and mood disorders (major depression and dysthymia). Fifty interviewers interviewed the subjects around, on average, 15.7 and 21.4 years of age.

#### 4. Personality traits

##### a. Diagnostic Assessment of Personality Pathology-Basic Questionnaire (DAPP-BQ) [19,34]

This two-hundred-ninety-question scale measures 69 specific, 18 basic, and 4 broad personality traits. The eighteen subscales consist of 12 to 16 questions about general personal preferences and behaviors. Internal consistency coefficients of those personality traits that were used in this study were acceptable (83–93).

##### b. Barratt Impulsiveness Scale [35]

This is a 30-item self-report instrument assessing trait impulsivity and referring to the general ways in which a person acts or thinks in different situations. The internal consistency in BIS-10, the version used in this study, was acceptable (α = .81).

#### 5. Suicidality

##### a. Violence of the method

Hanging, jumping from a high place, shooting a firearm, cutting, burning, and jumping under a vehicle were classified as violent suicide attempts. Non-violent methods encompassed drug overdose, drowning, carbon monoxide poisoning, and induction of hypothermia through the use of alcohol and neuroleptic drugs [36].

##### b. Scale for Suicidal Ideation [37,38]

This 19-item self-report scale consists of four sections measuring current attitudes toward living and dying, characteristics of suicidal ideation, and actualization of contemplated attempt. The previously reported scale reliability varied between .89 and 92. Our Cronbach alpha was .63.

##### c. Suicide Intent Scale (SIN) [39]

This 20-question scale is administered to self-reported suicide attempters and designed to assess the "intensity" of the wish to die during their most recent attempt by measuring thoughts and feelings and objective circumstances at that time. Alpha coefficient in the present study was .64.

##### d. Suicide attempts (lifetime)

A three-level outcome variable, classifying individuals into non-attempters, one-time and multiple suicide attempters, was created using information extracted from several scales and at two assessment waves:

i) Early adulthood:

-"Have you already attempted suicide?" (SIN)

-"How many attempts have you made prior to the one in question?" (SIN)

ii) Midadolescence

The presence and frequency of suicide attempts over lifetime and six-months were assessed using parental and adolescent responses to three questions originating in DISC-2 scales, designed to screen for depressive symptomatology:

-"Have you already attempted suicide?"

-"How many times?"

-"Have you attempted suicide during the last 6 months?"

##### e. Suicidal ideation (lifetime)

A three-level outcome variable was created using information extracted from three different scales in adolescence and early adulthood, with suicidal ideas being reported once classified as transient and those reported on at least two occasions as persistent.

#### 6. Social support

##### a. Social Support Scale [40]

This instrument measures perceived support by friends and family using fifteen statements. Item response levels range from 'not at all like me' to 'a lot like me'. Cronbach alpha was .88.

### Statistical procedures and analytical approach

Identification of the correlates of suicide attempts proceeded in several steps. We began with univariate analyses (ANOVAs, Scheffe Multiple Comparison, and Chi square tests) to pre-select variables for inclusion in multivariate analyses, focusing on several domains previously shown to be associated with our outcomes of interest: sociodemographic (parental socioeconomic status, education and ages at birth of first child, gender, and participants' adult income), psychiatric (adolescent and adult Axis I disorders), experiential (physical and sexual abuse, social support, stressful events), and 17 narrow and 4 broad personality traits.

We adhered to the model-building logistic regression protocol outlined by Hosmer & Lemeshow [41]. Significant main effects within each domain of risk factors were identified in a series of hierarchical regression analyses, beginning with the univariately-significant demographic variables. Multinomial logistic regression was used to identify the best model for a three-level outcome ("2" = repeated suicide attempters, "1" = one-time suicide attempters; "0" = non-attempters). We built two multinomial models, using either broad or narrow personality traits, along with the univariately-significant variables from other risk domains. Each model was adjusted for a possible confounding effect of the childhood mood disorder as it was not completely independent of the response variable (see Methods, Suicidality subsection).

We compared the two models' overall success in identifying correlates of one-time and repeated suicide attempts using Bayesian (BIC = -2LL + ln(N) × k) and Aikaike Information Criteria (AIC = -2LL + 2 × k), where N stands for sample size, ln for natural logarithm, k for number of parameters, and LL for log likelihood.

Weighted analyses were adjusted by the inverse of the participant's probability to be missing on the outcome. This probability was conditioned on family's socioeconomic status and participant's gender- variables that were related to attrition in our cohort. Since weighted analyses yielded similar results to our unweighted analyses, only the former are presented.

## Results

### Univariate and descriptive statistics (Table 1; Figures 1 and 2)

**Figure 1:**
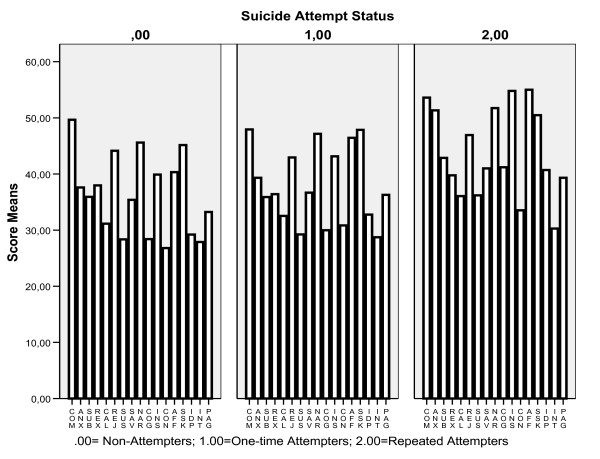
**Narrow Personality Trait Scores in Nonattempters, One-time and Repeated Attempters**. com = compulsivity, anx = anxiousness, sub = submissiveness, rex = restricted expression, cal = callousness, rej = rejection, sus = suspiciousness, nar = narcissism, cog = cognitive-perceptive dysregulation, ins = insecure attachment, con = conduct problems, aff = affective instability, ssk = stimulus seeking, idp = identity problems, int = intimacy problems, pag = passive aggressivity.

**Figure 2:**
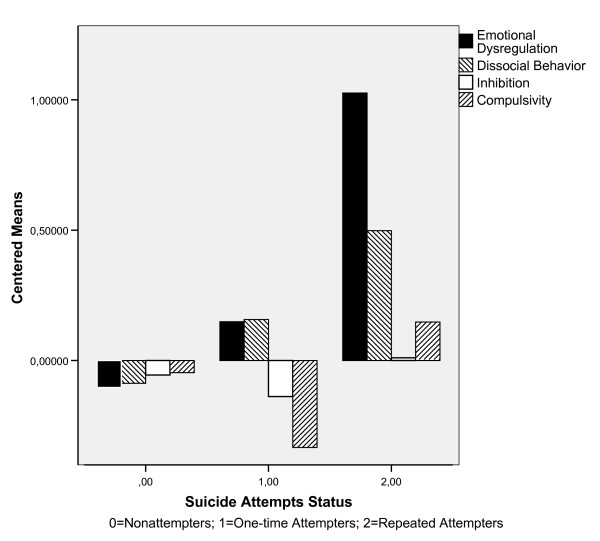
Broad Personality Trait Scores in Nonattempters, One-time and Repeated Attempters.

**Table 1:** Univariate personality trait differences between nonattempters, one-time and repeated suicide attempters

	**Nonattempters (NSA)**		**One-time Attempters (SA)**		**Repeated Attempters (RSA)**	
	
	**Mean**	**SD**	**Mean**	**SD**	**Mean**	**SD**
Emotional Dysregulation	-.07b***	.98	.18^c^***	.95	1.07^b***c***^	1.08
Dissocial Behavior	-.03^b**^	.99	.23	1.10	.58^b**^	1.27
Inhibition	-.01	.97	-.10	1.15	.07	1.12
Compulsivity	-.01^a&^	1.01	-.30^a&c&^	.94	.19^c&^	.94
Affective instability	40.33^a***b***^	11.85	46.45^a***c**^	12.08	55.00^b***c**^	12.11
Compulsivity	49.65	11.00	47.93	11.08	53.60	9.81
Conduct problems	26.81^a**b***^	8.51	30.83^a**^	10.33	33.53b***	10.72
Submissiveness	35.94^b**^	9.89	35.89^c**^	9.67	42.87^b**c**^	8.88
Cognitive distortion	28.39b***	9.20	29.97^c***^	9.00	41.20^b***c***^	13.18
Identity problems	29.21^a*b***^	11.02	32.76^a*^	11.97^c**^	40.40 ^b***c**^	15.49
Stimulus seeking	45.15	11.72	47.87	11.57	50.47	11.10
Callousness	31.14^b*^	8.80	32.54	10.95	36.07^b*^	12.85
Rejection	44.14	10.48	42.95	10.44	46.93	11.84
Insecure attachment	39.87b***	13.62	43.15^c***^	15.07	54.80^b***c***^	11.88
Anxiousness	37.60b***	12.63	39.33^c***^	12.04	51.33^b***c***^	14.13
Suspiciousness	28.35b***	8.81	29.24 ^c***^	9.32	36.20^b***c***^	11.31
Social avoidance	35.39^b*^	11.99	36.67	13.01	41.00^b*^	11.59
Narcissism	45.59^b*^	12.09	47.16	12.48	51.73 ^b*^	12.85
Restricted expression	37.97	10.52	36.41	11.32	39.77	10.13
Passive aggressivity	33.21^a*b**^	9.79	36.29^a*^	11.17	39.33^b**^	12.79
Intimacy problems	27.89	8.71	28.72	10.41	30.27	9.97

**Notes: **Procedure: Scheffe multiple comparison test; SD = standard deviation;^&^= statistical trend,* .05–.01; ** .01 to.001, inclusive; ***.0005; Statistically significant differences between ^a ^NSA and SA,^b ^NSA and RSA, and ^c ^SA and RSA groups.

Figure 1 shows narrow personality traits scores to be the highest in repeated suicide attempters. The exceptions were scores in three of these traits: compulsivity, rejection and restricted expression (Table 1). ANOVA-based analyses demonstrated significant differences between the three subgroups in 13 narrow personality traits at p = .05 level and in 9 of them at Bonferroni-corrected p value (p = .002; 21 tests): [anxiousness (F_2,897 _= 17.42; p = .0005), submissiveness (F_2,897 _= 7.20; p = .001), suspiciousness (F_2,897 _= 11.27; p = .0005), cognitive-perceptive dysregulation (F_2,897 _= 27.71; p = .0005), insecure attachment (F_2,897 _= 18.58; p = .0005), conduct problems (F_2,897 _= 14.97; p = .0005), affective instability (F_2,897 _= 29.74; p = .0005), identity problems (F_2,897 _= 17.71; p = .0005), and passive aggressivity (F_2,897 _= 8.11; p = .0005)]. The Scheffe multiple comparison test identified several narrow traits differentiating between repeated suicide attempters and the remaining two groups (submissiveness, cognitive-perceptive dysregulation, insecure, suspiciousness, and anxiousness); between suicide attempters and nonattempters (conduct problems and passive aggressivity), and among all three groups (affective instability and identity problems).

Broad personality trait profiles were quite different across nonattempters, first-time attempters and repeaters (Figure 2). ANOVA tests showed significant differences in three of these traits: Emotional Dysregulation (F_2,897 _= 21.51; p = .0005), which also differentiated between repeated suicide attempters and the remaining two groups (Table 1), Dissocial Behavior (F_2,897 _= 7.26; p = .001), and Compulsivity (F_2,897 _= 3.70; p = .025). Overall, variability in scores among nonattempters was very low (Figure 2). Nonattempters also had lower Emotional Dysregulation and Dissocial Behavior than both groups of suicide attempters. Relative to nonattempters, Compulsivity and Inhibition scores were higher in repeated and lower in one-time suicide attempters. Repeated suicide attempters had the highest levels of all four broad personality factors (Figure 2).

### Multivariate statistics: multinomial regression models (Tables 2 and 3)

**Table 2:** Suicide attempts and broad personality traits: a multinomial regression model

	**Likelihood of RSA vs NSA**			**Likelihood of SA vs NSA**			**Likelihood of RSA vs SA**		
	B (SE)	Exp [B](95% CI)	P	B (SE)	Exp [B](95% CI)	P	B (SE)	Exp [B](95% CI)	P

Childhood mood disorder	1.23(.62)	3.43(1.02–11.54)	.047	.19(.51)	1.20(.44–3.30)	.720	-1.05(.69)	2.85(.74–10.94)	.128
Childhood sexual abuse	.40(.10)	1.49(1.23–1.81)	.0005	.15(.09)	1.16(.97–1.38)	.096	.25(.11)	1.29(1.03–1.61)	.025
Suicidal ideation (lifetime)	2.13(.42)	8.39(3.71–18.99)	.0005	1.54(.20)	4.67(3.18–6.86)	.0005	.59(.44)	1.80(.75–4.29)	.187
Compulsivity	.47(.25)	1.60(.97–2.62)	.063	-.23(.15)	.79(.59–1.06)	.125	.70(.28)	2.01(1.17–3.45)	.011

Model fit: *X*^2 ^_8 _= 159.25 p = .0005;McFadden R^2 ^= 27.8%; RSA = Repeated suicide attempters (n = 34); SA = one-time suicide attempters (n = 90); NSA = nonattempters (n = 970).

**Table 3:** Suicide attempts and narrow personality traits: a multinomial regression model

	**Likelihood of RSA vs NSA**			**Likelihood of SA vs NSA**			**Likelihood of RSA vs SA**		
	B (SE)	Exp [B](95% CI)	P	B (SE)	Exp [B](95% CI)	P	B (SE)	Exp [B](95% CI)	P

Childhood mood disorder	1.01(.62)	2.75(.815–9.28)	.103	.19(.52)	1.20(.43–3.36)	.723	.83(.69)	2.29(.59–8.91)	.234
Childhood sexual abuse	.37(.10)	1.45(1.20–1.77)	.0005	.11(.09)	1.12(.93–1.33)	.230	.27(.12)	1.30(1.04–1.64)	.023
Suicidal ideation (lifetime)	1.89(.43)	6.61(2.86–15.27)	.0005	1.81(.23)	6.10(3.90–9.54)	.0005	.08(.46)	1.08(.44–2.68)	.863
Anxiousness	-.01(.03)	.99(.93–1.05)	.738	-.09(.02)	.92(.88–.95)	.0005	.08(.03)	1.08(1.01–1.15)	.024
Affective instability	.05(.03)	1.05(.99–1.12)	.117	.07(.02)	1.08(1.04–1.12)	.0005	-.02(.03)	.98(.91–1.05)	.501

Model fit: *X*^2 ^_10 _= 178.48 p = .0005; McFadden R^2 ^= 31.2%; RSA = Repeated suicide attempters (n = 34); SA = one-time suicide attempters (n = 90); NSA = nonattempters (n = 970).

In the first model, which featured broad personality traits, Compulsivity was the only significant correlate, differentiating between repeated and one-time suicide attempters [(OR = 2.0; 95% CI 1.2–3.5)]. In the second model, two narrow traits made significant contributions and had similar effect sizes. Both affective instability [(OR = 1.1; 95% CI 1.04–1.1)] and anxiousness [(OR = .92; 95% CI .88–.95)] differentiated between nonattempters and one-time attempters Anxiousness also differentiated between repeated attempters and one-time suicide attempters [(OR = 1.1; 95% CI 1.01–1.1)].

In both models, suicidal ideation and CSA (childhood sexual abuse) had positive associations with suicide attempts. CSA effect size was similar in both models [(OR = 1.5; 95% CI 1.2–1.8)]. In contrast, suicidal ideation had a greater range of effect sizes (ORs = 4.7–8.4) in the broad than in the narrow personality trait model (ORs = 6.1–6.6). In terms of the quantitative differences, according to our information criteria, the narrow personality trait model [AIC = 412.878; BIC = 468.958] performed somewhat better than the one featuring broad traits [AIC = 433.669; BIC = 480.403].

## Discussion

Up to a half of all suicide attempters, according to some estimates, may have made more than one suicide attempt in their lifetime [42,43]. Compared to one-time suicide attempters, repeaters are more likely to complete suicide [44,6]. They also have more severe clinical and behavioural profiles, including more extensive emotional regulation problems, aggression, impulsivity, cognitive rigidity, and neuroticism [16,6,45,46].

Establishing which personality traits have the potential to differentiate between individuals engaging in suicidal acts of different degrees of severity would help us to identify persons at risk and also to understand the processes underlying the link of personality and nonfatal suicidal behaviours. The present study makes a unique contribution to the field by investigating qualitative and quantitative differences between broad and narrow personality trait correlates of suicide attempts. Our findings suggest that anxiousness and Compulsivity may successfully differentiate between repeated and one-time suicide attempters. Anxiousness and affective instability, on the other hand, may be useful in differentiating between nonattempters and one-time suicide attempters.

### Personality trait profiles in repeated and one-time attempters

Our univariate findings suggest that, relative to one-time attempters and nonattempters, multiple suicide attempters score higher on many of the personality traits measured in this study. A broad personality trait defined as Emotional Dysregulation as well as five of its cognate narrow personality traits, appear to be markers of repeated suicide attempts. Two additional traits related to this broad dimension differentiated between all three groups of participants in a dosage-dependent fashion, being the highest among repeated attempters.

Emotional Dysregulation is related, although not identical, to Neuroticism and Harm Avoidance [9,19,47,48]. It consists of the shared variance of ten narrow personality traits affecting self-perception (narcisissm, identity problems), interpretation of a situation (cognitive perceptive dysregulation), reaction to perceived stressors (anxiousness, affective instability, passive-aggressivity), and interaction with social environment (submissiveness, insecure attachment, social avoidance, and suspiciousness). Because it affects multiple, and essential, aspects of functioning, it is not surprising that, at least in persons with predisposition to self-harm, Emotional Dysregulation may lead to repeated suicide attempts [16]. More specifically, repetition may be related to one's inability to tolerate and express frustration, on the one hand, and, on the other, to one's perception that a suicide attempt is "an immediate, constantly available, effective recourse at times of crises" [49].

### Broad and narrow personality traits as markers of suicide attempt status

In answer to our main research question – which of two levels of personality traits may be more useful as markers of different subgroups of suicide attempters- we identified both qualitative and quantitative differences. In statistical terms, the model featuring narrow personality traits performed better. Nevertheless, the associated effect sizes were bigger for the broad than for the narrow personality trait correlates.

Concerning qualitative differences, the final, narrow personality trait model included affective instability and anxiousness, components of the broad Emotional Dysregulation factor. The broad personality trait model, on the other hand, featured Compulsivity, factor consisting of the shared variance of the narrow traits of rejection and compulsivity. This broad factor is related to obsessive-compulsive personality disorders and borderline symptoms [50,51]. Obsessive-compulsive tendencies – "repetitive unpleasant thoughts and ritualized behaviors" [52]- have, in turn, been linked to suicide attempts and self-harm, although research on repeated suicide attempts is lacking [53-55]. In addition, repetitive self-harm and low-lethality suicide attempts are one of the main features of borderline personality disorder, possibly associated with impaired decision-making and cognitive rigidity [56]. The latter, along with interpersonal hostility, is one of the main facets of rejection, a trait underlying the Compulsivity factor. All three of these personality constructs have been linked to suicidal tendencies in diverse populations [57,58,24].

Specific, rather than only shared, variance associated with anxiousness and affective instability contributed significantly in the model examining narrow trait correlates of suicide attempts. Interestingly, the effects of these two highly correlated traits (r^2 ^= .70) were quite distinct. Affective instability levels were significantly higher in one-time suicide attempts than in nonattempters. While previous evidence in individuals who have attempted suicide once is lacking, positive associations of suicide attempts and constructs similar to affective instability have been reported before. For example, emotional instability is a correlate of completed suicide [59] and of borderline personality disorder, a recognized clinical risk factor of suicide attempts [50]. Facets of affective instability, such as irritability, have, furthermore, been found to be relevant in different subpopulations of suicide attempters: males [60], college students [61], and depressed patients [62].

Anxiety had a dual role: it was related to a lower likelihood of one-time suicide attempts but a higher likelihood of repeated suicide attempts. The second scenario -that anxiousness is more likely to be higher in repeated than in first-time suicide attempters -is in line with the finding that childhood anxiety predisposes to multiple suicide attempts [63]. Other studies have, for the most part, however, focused on suicide attempters as a group, without distinguishing between one-time and repeated attempters, typically finding it to be higher in suicide attempters of different age and clinical histories [6,64,65].

The first finding – that anxiousness may be associated with a somewhat lower likelihood of attempting suicide once rather than never- is not easily explained, although a similar scenario has been reported before. Low anxiety in different age groups increased the risk of suicide death [59,66]. Alternatively, this negative association may be related to the characteristics of the suicide attempt such as its timing. If recent, suicide attempts, for example, may have alleviated anxiousness [49]. Cathartic effects – a decrease in negative feelings following a suicide attempt [67,68]-have been observed for several pathological personality traits [49]. Personal interpretation and definition of "suicide attempts" may have also differed between self-reported one-time suicide attempters and nonattempters. Additionally, the relationship of anxiousness and suicide attempts may indeed be bi- or multimodal and differ across anxiety levels. Namely, slightly elevated anxiousness may not be as incapacitating as its excessively high levels and is possibly more conducive to help-seeking behaviors or better management of suicidal thoughts before they escalate into suicidal acts. Alternatively, an unidentified moderator may be involved [69,70]. Gender and sociodemographic factors are possible, although, judging by the evidence to date, unlikely candidates [71]. Our own gender-based moderating analyses were precluded by a small number of males among repeaters.

In conclusion, significant personality correlates in our two models are associated with two different personality dimensions: Compulsivity and Emotional Dysregulation. One of the explanations for this intriguing finding may be related to the fact that narrow and broad personality traits are not directly comparable as they are not structurally equivalent. As mentioned earlier, broad traits, such as Compulsivity, consist only of shared variance of their associated narrow traits. In contrast, the shared variance of anxiousness and affective instability, two of the narrow personality traits contributing to Emotional Dysregulation and to our second model, was controlled for in our regressions. This difference, however, does not invalidate their relevance nor previous support for the involvement of several different personality constructs in suicidal behaviors.

## Conclusion

Personality traits' influence on health outcomes is multidimensional and possibly mediated by socioenvironmental factors, health-related behaviours, and a number of psychophysiological processes [72,73]. In terms of the suicide attempts of different severity, our data demonstrated that both emotional and cognitive dysregulation may be involved.

Whether narrow or broad personality traits may be more optimal in differentiating between multiple and one-time suicide attempters depends on the specific research objective. Findings associated with narrow traits may be easier to interpret and link to existing known sociobiological theories. Larger effect sizes associated with broad traits may be better suited to objectives with a more clinical focus. Nevertheless, the extent to which these and similar conclusions are contingent on differences in sample characteristics, temporal changes, and statistical approaches requires further research consideration.

Most measures were retrospective and subject to recall bias. Psychometric properties of instruments assessing suicidal intent and suicidal ideation were not as high as expected, possibly due to translation problems. Nevertheless, our assessment of suicidality relied on a number of other measures in addition to these two scales.

We were also unable to exclude non-specific, cohort effects resulting from repeated assessments. We are, moreover, unsure of the extent to which the responses on personality measures were influenced by current states. Finally, our findings are based on a relatively culturally-homogeneous community sample and therefore have limited generalizibility to other cultural groups and to clinical populations.

## Competing interests

The author(s) declare that they have no competing interests.

## Authors' contributions

JB conceived of the study and statistical strategy, conducted data analysis and data interpretation, and drafted the manuscript.

RT coordinated and planned the psychosocial and clinical assessments and subject recruitment.

FV contributed to the planning of the psychosocial and clinical assessments and subject recruitment and provided input on data interpretation and editorial help.

MH contributed to the planning of the psychosocial and clinical assessments and subject recruitment.

JP provided editorial help and input on data interpretation.

GT supervised the study, provided feedback on the analyses and interpretation, and provided editorial help.

**All authors read and approved the final manuscript**.

## Pre-publication history

The pre-publication history for this paper can be accessed here:


